# Thermal Analysis and Selected Properties of CuNi2Si Alloy Used for Railway Traction

**DOI:** 10.3390/ma14164613

**Published:** 2021-08-17

**Authors:** Jarosław Konieczny, Krzysztof Labisz

**Affiliations:** Department of Railway Transport, Faculty of Transport and Aviation Engineering, Silesian University of Technology, 40-019 Katowice, Poland; krzysztof.labisz@polsl.pl

**Keywords:** alloyed copper, differential scanning calorimetry, elastomeric tests, corrosion resistance, abrasion resistance

## Abstract

This paper investigates the effect of high-temperature aging (600 °C and 650 °C) on the microstructure and functional properties of copper CuNi2Si alloy. The paper also presents the results of elastomeric tests performed by means of the Gleeble 3800 heat and plastic treatment simulator, as well as DTA (Differential Thermal Analysis) analysis carried out for the investigated alloy aged for 1, 2, 4 and 7 h. Corrosion resistance tests were performed by means of the potentiodynamic method with Atlas Sollich Atlas 0531 potentiostat/galvanostat in a 3% sodium chloride solution. Based on the tribological tests, it was confirmed that the CuNi2Si alloy was solution heat treated from the temperature of 1000 °C and gradually aged at the temperature of 600 °C and 650 °C for 1–7 h, characterized by a stable wear resistance. The alloy aged at the temperature of 600 °C was characterized by a lower mass loss compared to the one aged at 650 °C. Based on the DTA analysis, it was found that for the alloy aged for 2, 4 and 7 h, at the temperatures of 401 °C, 411 °C and 412 °C, respectively, the decomposition of a supersaturated solid solution took place by spinodal transformation accompanied by a sequence of phase transitions DO_22_ [(Cu, Ni)_3_Si],→ δ-Ni_2_Si → (Cu, Ni)_3_Si. The results of these investigations have proved that the CuNi2Si alloy can be widely used for electric traction. The use of alloys that replace elements made entirely of copper and, in this way influencing its lower demand, is in line with the global policy of economical management of natural resources.

## 1. Introduction

Among all the copper alloys, beryllium copper has the best strength properties, high electrical conductivity as well as resistance to corrosion and abrasion [1]. One of the most important advantages of these alloys is that they are non-sparking. However, copper alloys with the addition of beryllium are very toxic. The result of the search for alternative substitutes for CuBe alloys is, among others, CuNiSi alloys [2]. Compared to beryllium bronzes, they are characterized by similar electrical properties and comparable mechanical properties [3,4].

According to European Standards [5], there are 21 grades of alloyed copper, including three with the nickel concentration of 1.3%; 2.0% and 3.5% and the silicon concentration of 0.5% to 1.2%. Commercial applications of this grade of alloyed copper take advantage of its good hot and cold formability, good machinability and wear resistance. Due to its good resistance to atmospheric corrosion, it is used as outdoor products: clamps, fasteners, nuts and also as electrode holders, brackets for line welding electrodes, butt or spark welding dies and ball bearing baskets, friction pads, fuse clips, contact springs [6]. In addition, railway equipment, accessories for the upper electrical traction network [7], marine equipment and armature. The alloy with the nickel concentration of 1.6–2.5% and silicon 0.5–0.8% (CuNi2Si/CW111C) is the most commonly used one [8,9,10,11,12,13,14,15,16,17,18,19].

The CuNi2Si copper alloy is one of the alloys strengthened by precipitation hardening. Usually, the heat treatment of these alloys consists of supersaturation (900–1000 °C) and aging (400–600 °C). Sometimes these alloys are treated by a combination of heat treatment and cold plastic deformation (e.g., rolling) in the sequence of supersaturation–cold rolling–aging [20].

Aging at high temperatures (≥600 °C) is rarely used in the processing of CuNi2Si alloys, especially for more than 5 h [1]. The properties that are shaped by such treatment are important for industrial applications. The corrosion resistance in the open air and the tribological wear resistance after aging at 650 °C for more than 5 h are of particular importance.

Railway electric traction (conductors or guides of section insulators) are primarily exposed to atmospheric corrosion (including, depending on the latitude, large temperature fluctuations depending on the seasons, sometimes even in the range of −40–30 °C) as well as chemical corrosion due to the transported materials, which are very often in the form of a cloud of dust, induced by a passing train, as well as electrochemical corrosion due to the share of the flowing electric current. For these reasons, the tests of corrosion resistance using the electrochemical method seem justified.

The aim of this work is to investigate the influence of high-temperature aging on the stability of functional properties, such as corrosion resistance, abrasive wear resistance as well as the influence of temperature changes on mechanical properties and phase transitions.

## 2. Materials and Methods

The chemical composition of the alloy is shown in Table 1. The alloy was solution heat-treated at 950 °C for 1 h with cooling in water and then aged at 600 °C and 650 °C for 1, 2, 4 and 7 h.

The obtained samples were tested for corrosion resistance by means of the potentiodynamic method. The corrosion tests were carried out with the Atlas 0531 Atlas Sollich potentiometer/galvanostat (Atlas-Sollich, Rębiechowo, Poland). The research environment was an aqueous solution of 3% sodium chloride. A fresh solution was used each time. The measuring stand consisted of a test device (potentiostat), a corrosion vessel and a computer with the AtlasCorr controlling software (2016, version 2.24, Atlas-Sollich, Rębiechowo, Poland). The samples for corrosion tests were prepared by grinding on 500, 800 and 1200 μm /mm^2^ grit sandpaper, and then degreased in acetone. The test area for all samples was set as 1 cm^2^. The tests were repeated three times for each sample.

The corrosion resistance tests with the electrochemical method were carried out in two stages. In the first stage, the open circuit potential Eocp was determined in no-current conditions (free potential) for 1 h. The second stage consisted in determining the anodic polarization curves forcing a potential change in the anode and cathode direction every 1 mV/s.

Recording of the anodic polarization curves allowed to determine the characteristic values for the corrosion resistance: corrosion potential (E_corr_), corrosion current (i_corr_), polarization resistance (R_p_), potential for damage to the passive layer (puncture, E_pass_) and repassivation potential (E_repass_). The values of the current and the corrosion potential were determined by means of extrapolation and Tafel analysis in the AtlasLab program from Altas Sollich (2016, version 2.24, Atlas-Sollich, Rębiechowo, Poland).

The wear resistance tests were carried out by means of the Taylor Hobson tribometer. The counter-specimen in the form of a ball with 6 mm diameter was made from Al_2_O_3_ and the load was 10 N. Each measurement consisted of 5000 cycles to 7.2 mm. The total distance covered by the sample each time was 36 m.

Cylindrical samples with a diameter of ϴ = 10 mm × 12 mm were prepared for the elastomeric tests. These tests were performed by means of the Gleeble 3800 heat and plastic simulator manufactured by DSI (Dynamic System Inc., Poestenkill, NY, USA). The Gleeble 3800 simulator is equipped with a direct resistance heating system which maintains the chosen temperature with the accuracy of 1 °C. The thermocouples are welded to the sample and collect the temperature values of the tested sample, while the system with direct temperature registration by means of resistance heating reacts to its changes and properly controls the intensity of the electrical current and ensures the appropriate set temperature value. The Gleeble 3800 system is equipped with a hydraulic mechanical system that allows for exerting pressures of 200 kN during compression, which enables testing at a deformation rate ranging from 0.0001 to 200 s^−1^. Variable displacement transducers and strain gauges for measuring the pressure allow obtaining feedback which enables accurate execution and high repeatability of the set mechanical values of the planned technological process. In order to reduce friction, tantalum films were used between the surface of the sample and the anvil surfaces, while both surfaces were covered with nickel-based high-temperature grease. Cylindrical samples with a diameter of ϴ = 10 mm × 12 mm from the tested CuNiSi alloy were subjected to resistance heating under protective argon gas at the rate of 3 °C s^−1^ to the set temperature and heated for 30 s. The compression of the samples was carried out at the deformation rate of 0.1, 1 and 10 s^−1^ in the temperature range from 500 to 1000 °C.

Zeiss Supra 25 scanning electron microscope (SEM) was used to carry out the microstructure examination (Carl Zeiss, Oberkochen, Germany).

The thermal analysis was performed by thermal analyser LINSEIS STA PT-1600 (LINSEIS, Selb, Germany). The samples of ~200 mg weight were placed in a platinum crucible and heated at the rate of 10 °C/min in the air up to 1100 °C.

## 3. Results and Discussion

**Figure 1:**
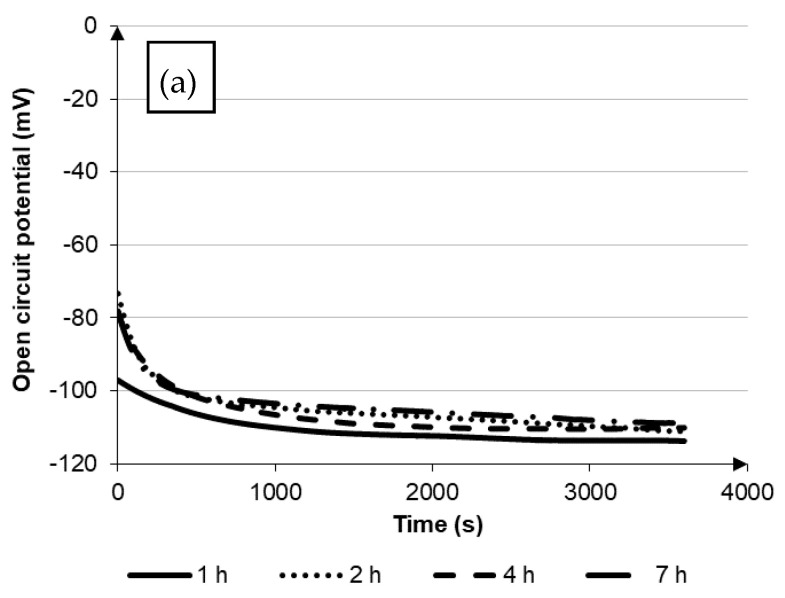

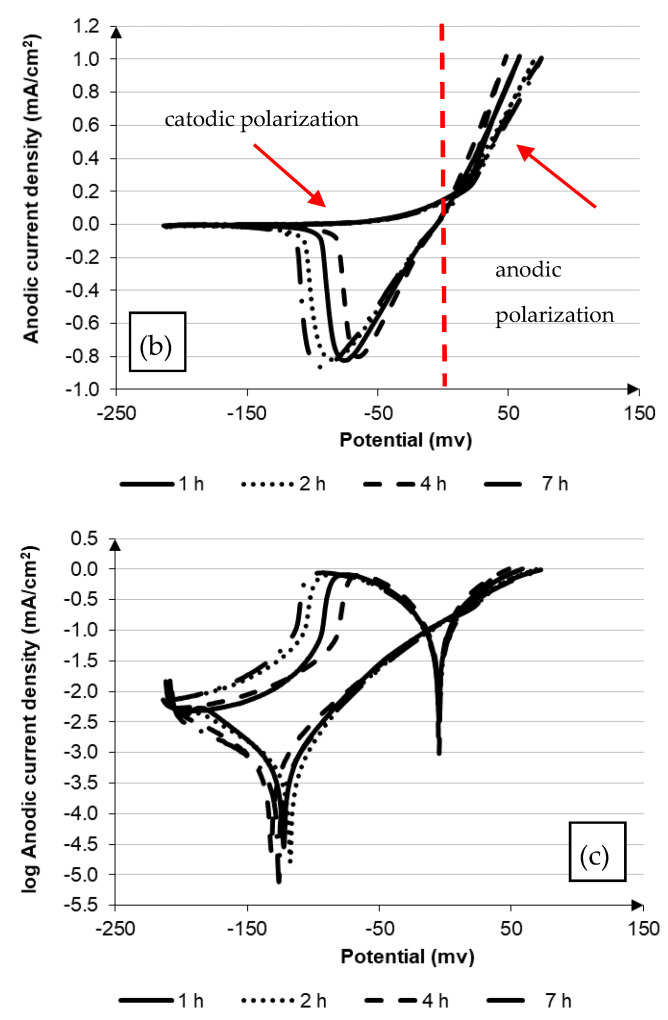
Electrochemical characteristics of the corrosion resistance test of aged samples at 600 °C: (**a**) potential of the open circuit, (**b**) anodic and cathodic polarization curve, (**c**) cycling potentiodynamic polarization curves with hysteresis loop (cathode and anode curves).

**Figure 2:**
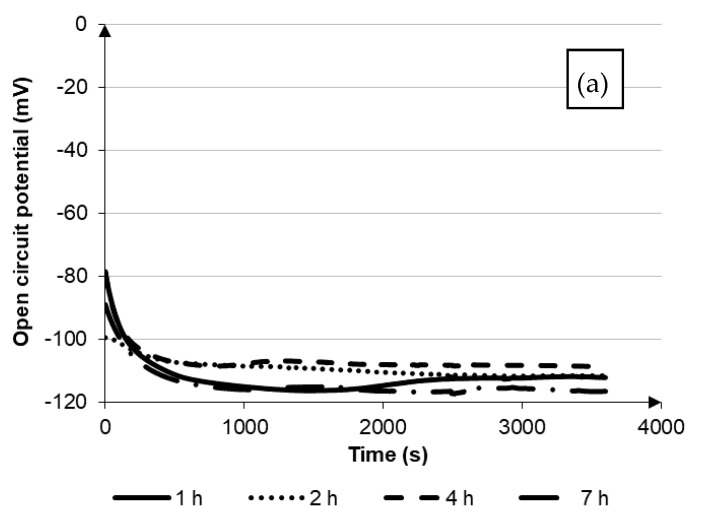

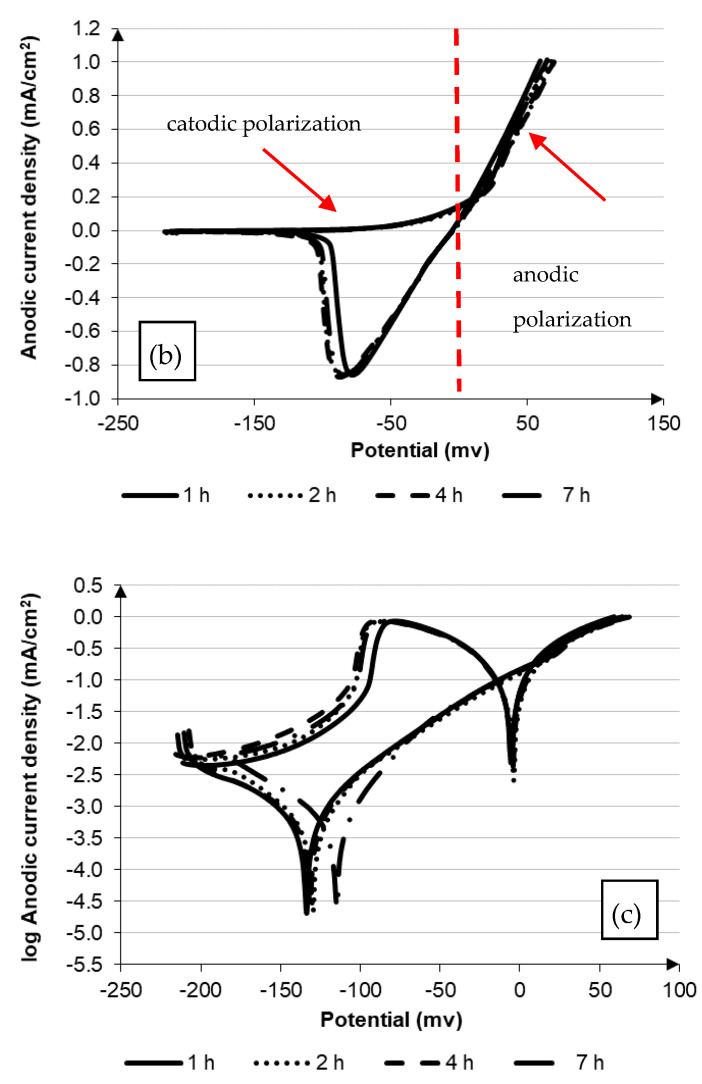
Electrochemical characteristics of the corrosion resistance test of aged samples at 650 °C: (**a**) open circuit potential, (**b**) anodic and cathodic polarization curve, (**c**) cycling potentiodynamic polarization curves with hysteresis loop (cathode and anode curves).

Copper and its alloys are characterized by excellent corrosion resistance in uncontaminated air and in drinking water at ambient temperature, the presence of chemical compounds or heat treatment may affect the change of this resistance [8,9]. The results of the corrosion resistance test CuNi2Si are presented in the form of functional dependencies E_ocp_ = f(t), I = f(E) as well as log(|i|) = f(E) in Figure 1 and Figure 2, whereas the values of the characteristic corrosion parameters are shown in Table 2.

Comparing the free potential values for the tested samples, which varied in a small range between −111 and 67 mV, it was found that it was difficult to indicate how the aging of the material affected the corrosion resistance. Although, based on the analysis of the results for samples aged at 650 °C, the value of open circuit potential increased together with the extension of the aging time. For the sample aged for 7 h, it almost doubled. For the samples aged at 600 °C, such a similar relationship was not observed. On the basis of the obtained results, it was found that for the alloy aged at 600 °C, together with the extension of the aging time, there was a positive direction shift, however, at 650 °C with the aging time of 7 h, the open circuit potential shifted towards even more negative values; and after 2 h of aging, there was a change in the shift of the open circuit potential, which was equal to the base value for the time over 2000 s for both curves.

With the forced change of potential, the values of corrosion potential and current were determined, which varied for both groups of samples tested in the same range. In the case of corrosive potential, its value was in the range from −122 to −99 mV and again only in the case of samples aged at 650 °C, it was possible to observe the relationship between the aging time and the value of the potential, which increased from −122 mV after 1 h of aging to −99 mV after 7 h. However, when analysing the influence of the aging time of the samples on the value of the corrosion current, for both samples aged at 600 °C and 650 °C it was found that the extension of time caused the occurrence of a higher current, and thus a greater dissolution of the surface of the material. By extending the aging time at 600 °C from 1 to 4 h, the current value almost doubled, and after extending the time to 7 h, it almost tripled. Similarly, in the case of aging at 650 °C, after extending the time to 4 h, the value of the corrosive current increased almost three times, while extending the time to 7 h increased its value almost 3.5 times. Lowering the corrosion resistance could be observed by comparing the values of the polarization resistance. It was found that after aging at 600 °C and 650 °C, the polarization resistance values were reduced by half after extending the aging time to four or more hours.

The change in the temperature and the aging time of the tested samples did not affect the breakdown potential of the passive layer, which was between 12 and 18 mV, and the value of the repassivation potential did not change and it oscillated around 6 mV in all conditions, except for the sample after aging at 600 °C for 1h for which the value of repassivation potential was 12 mV, i.e., twice as high.

**Figure 3:**
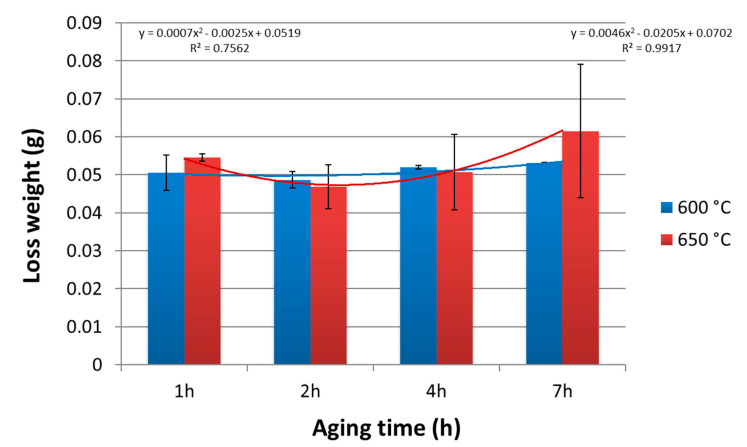
Comparison of tribological wear resistance of the CuNi2Si1 alloy solution heat-treated and aged for 1, 2, 4 and 7 h at 600 °C and 650 °C.

Based on the results of tribological tests, it was found that the CuNi2Si alloy solution heat-treated from the temperature of 1000 °C and then aged at 600 °C and 650 °C for 1, 2, 4 and 7 h was characterized by relatively stable wear resistance. At 600 °C, the difference in weight loss between the solution heat-treated state for 1 and 7 h was much lower than at 650 °C (Figure 3). The lowest values of weight loss were recorded for the condition after aging for 2 h for both temperature values of 600 °C and 650 °C. This was probably related to the process of precipitating the δ-Ni_2_Si phase particles [10,11] which was the effect of discontinuous precipitations in CuNiSi alloys [12,13]. These particles were responsible for the precipitation hardening effect in CuNi2Si alloys. The Ni_2_Si particles were released also at lower temperatures [14] and after a very short aging time. In the initial phase, after such a short aging time they reached the smallest diameter. Extending the aging time caused an increase in the size of δ-Ni_2_Si phase particles, with the use of the Orowan‘s mechanism, which contributed to the increase in strength enhancement [15] and wear resistance.

The approximation of the results of the tribological test for the CuNi2Si alloy aged at 600 °C showed that the aging time to weight loss relationship can be expressed by the equation:

(1) $$y=0.0007x^{2}-0.0025x+0.0519$$

coefficient of determination:

R^2^ = 0.7562 (2)

while for the aging temperature of 650 °C, this relationship can be determined by the equation:

(3) $$y=0.0046x^{2}-0.0205x+0.0702$$

coefficient of determination:

R^2^ = 0.9917 (4)

**Figure 4:**
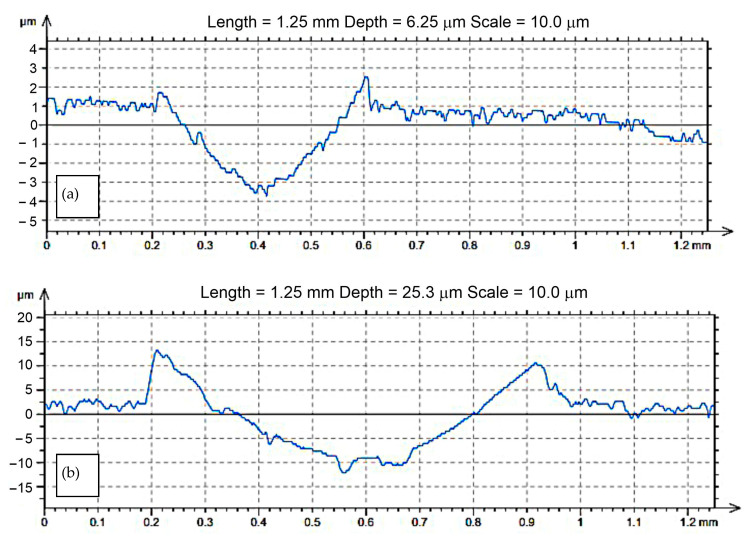
Transverse section of the scratch groove profile in the CuNi2Si alloy sample with indenter from Al_2_O_3_; (**a**) alloy supersaturated and aged at the temperature of 600 °C, (**b**) 650 °C for 60 min.

Additionally, the comparison of the cross-sectional shape of the scratch groove for the sample aged at 600 °C and 650 °C for 1 h showed that the plasticity of the alloy increased with the increase in temperature, which resulted in a wider and deeper groove (Figure 4).

**Figure 5:**
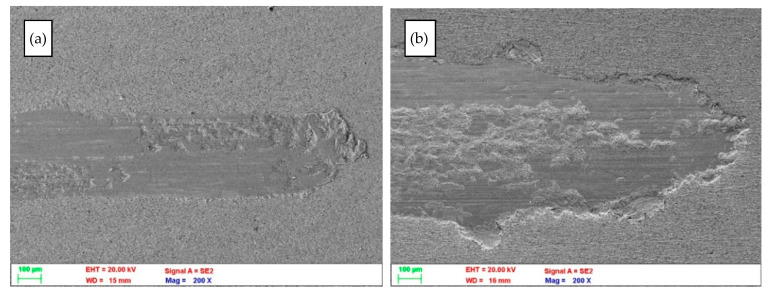
SEM images of the scratch trace of the surface of the CuNi2Si alloy made with an Al_2_O_3_ indenter, solution heat treated and aged at 650 °C for (**a**) 1 and (**b**) 7 h.

The abrasion trace of the CuNi2Si alloy surface is shown in Figure 5. Observations on the scanning microscope confirmed that even for an aging sample at the same temperature, increasing the aging time from 60 min to 7 h caused approximately a double increase in the width of the scratch groove. In addition, its character also changed.

**Figure 6:**
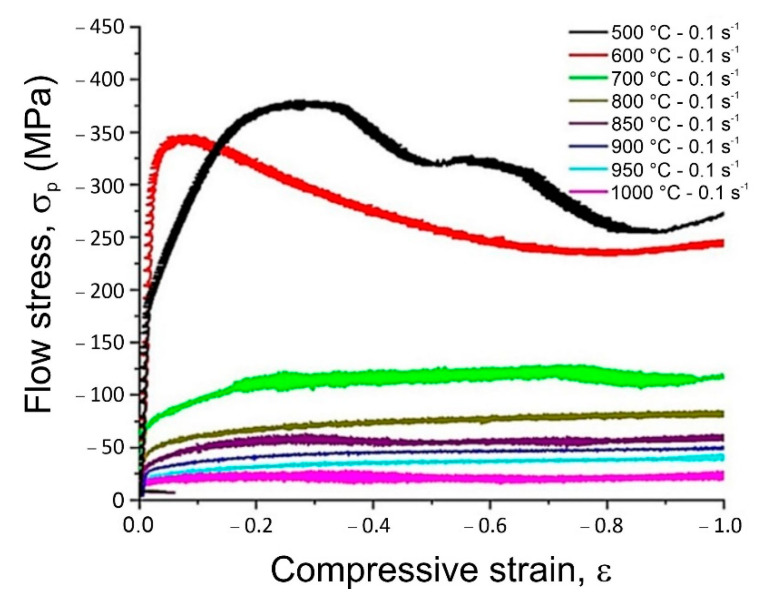
Effect of the strain temperature on the shape of stress–strain curves of the CuNiSi alloy after real deformation: ε = 1, with a constant strain rate equal to 0.1 s^−1.^

**Figure 7:**
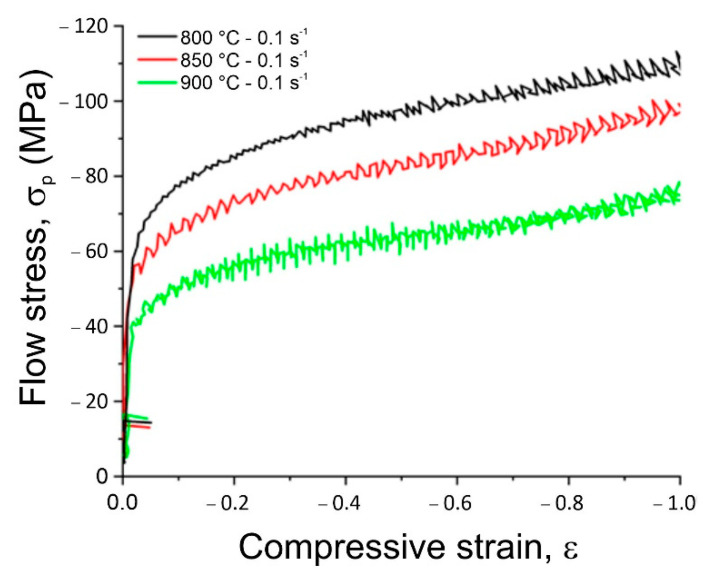
Influence of the strain temperature on the shape of stress–strain curves of the CuNiSi alloy after real deformation: ε = 1, at a constant rate of strain equal to 1 s^− 1^.

A detailed analysis of stress–strain curves for the tested CuNi2Si alloys indicated that the deformation temperature had a large influence on the value of the flow stress, which for the applied strain conditions assumed the values from 25 to 375 MPa for the samples deformed in the temperature range from 1000 to 500 °C respectively and the strain rate equal to 0.1 s^−1^ (Figure 6). A tenfold increase in strain rate to 1 s^−1^ caused that the values of the flow stress were in the range from 75 to 110 MPa for the samples already deformed only at temperatures of 900 °C, 850 °C and 800 °C, respectively. It was found that with the decrease in the plastic deformation temperature, the value of strain ε_max_ increased, corresponding to the maximum yield stress from 0.13 to 0.3 but only for the samples deformed at the rate of 0.1 s^−1^, that means that for these samples a dynamic recrystallization process was initiated. The increase in the strain rate to the value of 1 s^−1^ regardless of the change in the temperature of plastic deformation caused that the only process eliminating the effects of strain strengthening was dynamic recovery as evidenced by the shape of stress–strain curves (Figure 7). With the increase in plastic deformation, the stress values increased but they did not reach the maximum plasticizing stress ε_max_, and thus they did not reach the critical break which would allow the initiation of the dynamic recrystallization process during the plastic deformation at a given temperature. The strain rate 1 s^−1^ caused that all plastic deformation occurred within 1 s and this was too short to initiate dynamic recrystallization.

Based on the performed elastomeric tests of samples deformed at 0.1 s^−1^ and the results obtained in the form of stress–strain curves, it can be concluded that the plastically deformed sample at 500 °C after reaching a yield stress of 375 MPa at the deformation value of 0.3 cracked, which may mean that the temperature was too low and the sample was not sufficiently plasticized yet. The increase in the deformation temperature by 100 °C caused the reduction of the plasticizing stress to 350 MPa and already for the critical deformation of 0.08, the dynamic recrystallization was initiated. During further deformation of the test sample, the stress was reduced to about 240 MPa and the steady-state was reached. Increasing the plastic deformation temperature value for the next sample by 100 °C to 700 C caused a significant decrease in the stress to 120 MPa, while critical deformation was achieved for the deformation equal to 0.25. For the subsequent samples deformed at higher temperatures in the range from 800 °C to 1000 °C, it was found that the only process controlling strain strengthening in the entire temperature range of plastic deformation was dynamic recovery. So far, the elastomeric tests were only performed for the Cu_3_Ti alloys [22] and for the CuNiAl [23] alloys, therefore the results of the elastomeric tests of the CuNi2Si alloy will extend this knowledge.

**Figure 8:**
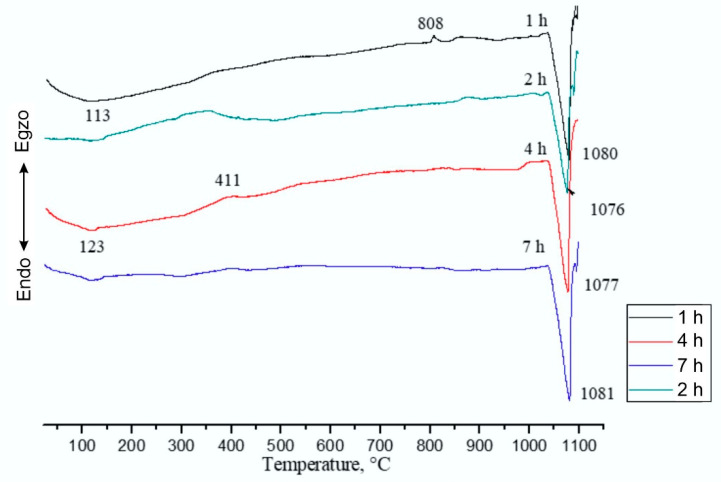
DTA curves of tested samples.

Figure 8 presents the DTA (Differential Thermal Analysis) analysis results for Cu–Ni–Si alloys. The temperature and form of peaks show that the aging of CuNi2Si copper alloy has influenced the functional properties.

DTA curves reveal that the shape of peaks and temperatures of endothermic and exothermic peaks maximums of samples change with the time of aging of CuNi2Si copper alloy.

The temperature of the first endothermic peak maximum is 113–123 °C is related to a significant loss of elastic resistance of high-strength copper-based alloys [16]. This effect is known as stress–relaxation and it usually leads to electrical contact problems because the contact forces are significantly reduced with time.

As it can be seen in Figure 8, the exothermic peak after 1h of aging corresponds to a solid/solid transition rather than a solid/liquid transition [17].

Another peak at the temperature of about 400 °C is related to the decomposition of a supersaturated solid solution, which proceeds through spinodal transformation [18]. It results in a microstructure consisting of alternately placed silicon-rich and silicon-depleted plates. In the initial stage of spinodal decomposition, the metastable ordered phase is precipitated with the structure DO_22_ ((Cu, Ni)_3_Si), from which the coherent δ-Ni_2_Si phase with the orthorhombic structure is then formed. The alloys hardening mechanism is ensured by the precipitation of the (Cu, Ni)_3_Si phase, the share of which in the alloy decreases with increasing aging time, and the δ-Ni_2_Si phase. The peak at 808 °C can be attributed to the continuous precipitation which occurs in CuNi2Si alloys at temperatures over 600 °C. Heat treatment at the temperature above 650 °C causes the separation of β-Ni_3_Si, δ-Ni2Si and δ′-Ni_2_Si particles [19].

The temperature of the last endothermic peak maximum (1076–1081 °C) is related to the melting point of pure copper [17]. With an increase in the aging time (1–7 h), the transformation occurs and the relevant temperatures are 1080 °C, 1076 °C, 1077 °C and 1081 °C, which are lower than the melting points of pure copper, β2-Ni_3_Si (1115 °C).

Based on the DTA analysis curves (Figure 8), it was found that the CuNi2Si alloy aged at 600 °C and 650 °C for 1–7 h was characterized by very high structure stability, and the transformations characteristic of this alloy took place at the temperature of about 1000 °C, which guarantees the stability of the structure and properties in the temperature range of electric traction operation, taking into account the temperature effect which is the Joule–Lenz effect resulting from the flow of electric current through the conductor.

Nickel in alloyed copper which occurs in the intermetallic phase plays a very important role in the basic alloy, therefore it is important to investigate the influence not only the heat treatment parameters, but also the chemical composition of the alloy and the structure and properties of the final product [24,25,26,27].

In the process of heat treatment with the intensity (speed) of changes, diffusion plays a decisive role. For this reason, the diffusion coefficient was calculated using the dependence of the reaction rate on the temperature, which is determined by the Arrhenius Equation (5):

(5) $$D_{\left(T\right)}=D_{0}exp\left(-\frac{Q}{RT}\right)$$

where *D*_(*T*)_—the diffusion coefficient at a specific temperature, *D*_0_—the pre-exponential factor, *Q*—the activation energy, *R*—the gas constant, *T*—the absolute temperature.

**Figure 9:**
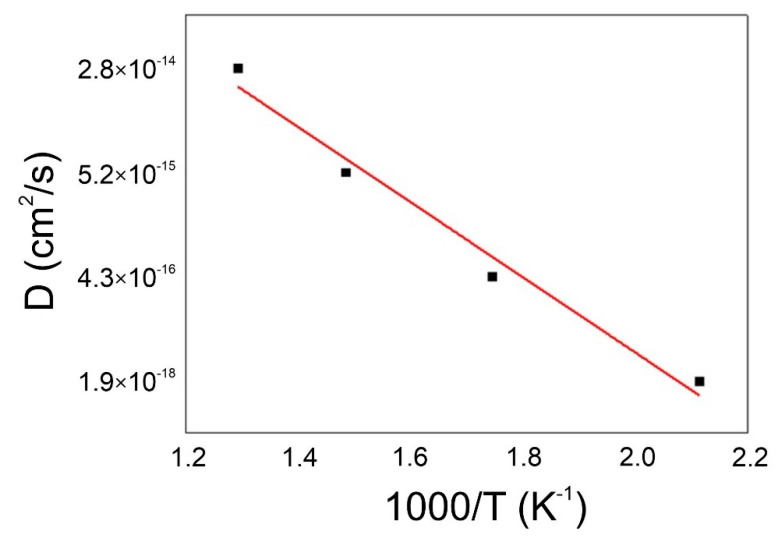
Arrhenius plot for D_c_ of CuNi2Si.

The influence of the aging temperature on the diffusion coefficient in CuNiSi alloys depends on the concentration of nickel and silicon in the alloy. The diffusion increases with increasing the temperature for all alloys with a nickel concentration in the range of 1.5–5.4% Ni. Figure 9 shows an Arrhenius plot for an alloy with the composition CuNi2.08Si0.89.

The obtained results of elastomeric tests revealed a number of processes taking place in the structure of the material depending on the temperature and rate of deformation, such as dynamic recovery and dynamic recrystallization, the knowledge of which has an application-related impact. In addition, the use of elements that are characterized by higher resistance to tribological wear also contributes to the implementation of the policy of economical management of natural resources and eliminates (to some range) the need to incur costs related to waste management.

## 4. Conclusions

As a result of the research, it was shown that at elevated temperatures (600 and 650 °C), the CuNi2Si alloy, which is characterized by stable tribological wear resistance, high resistance to atmospheric corrosion and structural stability, may be a potential replacement for Cu-ETP copper for railway traction cables.

There are various combinations of the Ni_x_Si_y_ phases in the isothermal section of the Cu-Ni-Si alloy. In the investigated alloy the Ni_2_Si two-phase region consisting of the FCC (face-centered cubic) Cu-alpha phase and the Ni_2_Si, as the dominant phase, is widened with an increase in the aging temperature from 600 °C to 650 °C. An increase in the aging temperature and time leads to the occurrence of spinodal decomposition during aging and decreases the diversity of the occurred phases, which, due to their different mechanical properties, increases the wear resistance of the alloy. Moreover, the wear resistance decreases due to the fact that the area of the FCC + Ni_2_Si phase in the copper-rich section of the Cu-Ni Si ternary alloy gets wider, so not only does the type of phase change but also the area of its occurrence.

The results of the corrosion resistance tests of the CuNi2Si alloy justify the use of these alloys as substitutes for the commonly used Cu-ETP or CuAg0.1 copper, which is characterized by more favourable mechanical properties and better resistance to tribological wear.

The CuNi2Si alloy, after high-temperature heat treatment, is resistant to tribological wear, which is shown by a very low weight loss, especially after the aging at 600 °C. The obtained results of microscopic analyses (SEM) confirmed that in the process of tribological wear of the CuNi2Si alloy, only a small volume of “waste” was produced.

As a result of the carried out research, it was found that after aging at 650 °C, the value of the open circuit potential increased with the extension of the aging time. For a sample aged 7 h, it almost doubled. For samples aged at 600 °C, no similar relationship was observed.

Based on the tests carried out, it was found that:

1. The value of open circuit potential for the CuNi2Si alloy aged at 650 °C increased with the extension of the aging time and for the sample aged for 7 h, this value almost doubled. Similar relationships were not found for the alloy aged at 600 °C. In the case of corrosive potential, again only for the alloy aged at 650 °C could be seen that the potential value increased with the extension of the aging time. The change in the temperature and aging time in the tested range did not affect the breakdown potential of the passive layer, and the value of repassivation potential did not change.
2. Based on the results of tribological tests, it was found that the CuNi2Si alloy solution heat treated from the temperature of 1000 °C and then aged at 600 and 650 °C for 1, 2, 4 and 7 h was characterized by fairly stable wear resistance. Increasing the aging temperature from 600 to 650 °C resulted in a reduction of wear resistance.
3. On the basis of performed elastomeric tests, it was found that the deformation temperature had a large impact on the flow stress value, which was equal for the applied deformation conditions 25–375 MPa for samples deformed in the temperature range from 1000 to 500 °C respectively by a strain rate of 0.1 s^−1^. It was also observed that as the temperature of plastic deformation decreased, the deformation value ε_max_ increased, which means that for these samples a dynamic recrystallization process was initiated.
4. Dynamic recrystallization was initiated for the deformation temperature of 600 °C and the critical pressure value of 0.08, For subsequent samples deformed at higher temperatures of 800–1000°C, it was found that the process controlling deformation in the entire temperature range of plastic deformation was the dynamic recovery.
5. The carried out DTA analysis confirmed that for the CuNi2Si alloy aged for 2, 4 and 7 h, the supersaturated solid solution decomposed at 401 °C, 411 °C and 412 °C, respectively. The mechanism of transformation is the so-called spinodal decomposition, accompanied by a series of phase transitions DO_22_ ((Cu, Ni)_3_Si) → δ-Ni_2_Si → (Cu, Ni)_3_Si. In addition, a continuous precipitation peak was observed for the alloy aged for 1 h at 808 °C. Heat treatment at temperatures above 650 °C caused precipitation of the β-Ni_3_Si, δ-Ni_2_Si and δ′-Ni_2_Si phase particles. The analysis of the results showed that for subsequent samples deformed at higher temperatures in the range from 800 °C to 1000 °C, the only process which determined the deformation strengthening in the entire plastic deformation temperature range was the so-called dynamic recovery. Based on the analysis of the DTA curves, it was found that with an increase in the aging time (1–7 h), the transformation occurred, and the relevant temperatures were 1080 °C, 1076 °C, 1077 °C and 1081 °C, which are lower than the melting points of pure copper, β2-Ni_3_Si (1115 °C).

## Figures and Tables

**Table 1:** Chemical composition of the tested CuNi2Si1 alloy [21].

Cu	Si	Zn	P	Pb	Sn	Mn	Ni	Sb	Bi	As	Cd
97.02	0.89	0.13	0.065	0.003	0.009	0.030	2.08	0.001	0.001	0.001	0.001

**Table 2:** Results of the corrosion resistance test of samples after aging.

Temperature	Time (h)	E_ocp_ (mV)	E_corr_ (mV)	i_corr_ (μA/cm^2^)	R_pcalc_ (kOhm/cm^2^)	E_pass_ (mV)	E_repass_ (mV)
600 °C	1	−78	−108	1.29	10.1	13	12
	2	−92	−122	1.36	10.3	14	7
	4	−90	−116	2.30	5.2	12	5
	7	−99	−112	3.15	5.01	16	6
650 °C	1	−111	−122	0.68	19.1	13	6
	2	−92	−109	1.08	16.6	18	−
	4	−74	−105	1.89	6.3	12	5
	7	−67	−99	2.29	5.2	12	5

## Data Availability

Data available on request.
